# Perinatal Mortality Due to Pre-Eclampsia in Africa: A Comprehensive and Integrated Approach Is Needed

**DOI:** 10.9745/GHSP-D-16-00054

**Published:** 2016-06-20

**Authors:** Moshood Omotayo, Katherine Dickin, Rebecca Stolzfus

**Affiliations:** a Cornell University, Ithaca, NY, USA

See related article by Hodgins.

In a recent *Global Health: Science and Practice* editorial, Hodgins identified key steps to “take a big chunk out of the wedge of maternal, newborn, and stillbirth mortality attributable to eclampsia/pre-eclampsia,” calling for early case identification with more frequent antenatal care (ANC) contacts, effective management of complications in pregnant women that progress to life-threatening states, and timely delivery.^1^

The editorial is an important call to action and we agree with its proposals; however, there are complementary programmatic issues that must also be considered in crafting an integrated strategy for addressing the burden of perinatal morbidity and mortality attributable to hypertensive disorders in pregnancy in Africa. These include: (a) strengthening management of preterm neonates, (b) strengthening delivery of secondary prevention through ANC, and (c) integrating primary prevention into ANC delivery.

## STRENGTHENING PRETERM MANAGEMENT

Effective surveillance and timely delivery as a strategy to address the burden of pre-eclampsia will likely increase incidence of medically indicated preterm births. Without concomitant action to strengthen the system to care for babies born too soon, the reduction in perinatal mortality might be limited. Preterm birth remains the single leading cause of neonatal mortality globally,^2^ and preterm infants are at greater risk of morbidity and neurodevelopmental delay. Despite evidence of low-cost measures that can reduce the scourge of this condition, adoption of these measures has been limited in low-income countries,^3^ leading to unacceptably high numbers of newborn deaths attributable to preterm births. Although studies have shown that increases in medically indicated cesarean deliveries and iatrogenic preterm births have been associated with reduction in maternal and neonatal mortality,^4^^,^^5^ this has been in the context of health systems with capacity for effective management of preterm neonates.

## STRENGTHENING DELIVERY OF SECONDARY PREVENTION THROUGH ANC

We agree with Hodgins that revisiting the policy on the recommended number of ANC visits in the third trimester is important, and we furthermore suggest that addressing current challenges and quality issues in pre-eclampsia screening and diagnosis in primary health care facilities is essential. Improving community demand for ANC should be emphasized. Regular and accurate blood pressure and proteinuria measurement in ANC is still the mainstay of pre-eclampsia detection and surveillance, but there are important coverage and quality gaps in these procedures in Africa, even when women present for ANC. Only 57% of pregnant women who had up to 4 ANC visits had urine samples collected, according to Demographic and Health Survey (DHS) data from 29 African countries (calculated from Hodgins 2014^6^). This represents an upper limit of the proportion of women that had urinary protein tests. Although an average of 78% coverage of blood pressure measurement was reported in the same analyses, studies in Africa have reported high levels of digit bias and other observer errors among nurses.^7^^,^^8^ In addition to assuring supply of functional sphygmomanometers and urinalysis testing kits, it is necessary to provide preservice and in-service training, continuous supportive supervision, and incentives to improve relevant skills for screening and diagnosis of hypertensive disorders in pregnancy in primary health care facilities.

## INTEGRATING PRIMARY PREVENTION INTO ANC DELIVERY

Integrating programs for primary prevention of pre-eclampsia into ANC, i.e., preventive calcium supplementation and low-dose aspirin in pregnancy, is essential. Primary preventive programs hold the promise of reducing the *incidence* of pre-eclampsia, without the risk of increasing the burden of preterm delivery, particularly in communities with inadequate dietary calcium intake, as is the case in many African communities.^9^ Integration of calcium supplements and low-dose aspirin into essential commodity supply chains, preservice and in-service training, supervision of health care workers in primary health care facilities, and community-based ANC programming all are critical for coverage, quality, and utilization of primary preventive programs. Although there are important clinical and implementation research issues that still need to be resolved for optimal calcium supplementation, functional programs can be designed and implemented with what is known.^10^ Strong preventive programming alone will address only a fraction of the problem,^11^ but it is a vital component of the mix because it reduces the need for more expensive lifesaving interventions requiring higher-level personnel.

By integrating primary preventive programming with strengthened capacity for case detection, timely patient transfer and delivery, and effective management of preterm neonates, maternal, stillbirth, and perinatal health indicators can be improved simultaneously.
